# Use of Nonconventional Sample Matrices for Endocrine Studies of Pit Vipers: Assay Validation and Applications

**DOI:** 10.1093/iob/obaf048

**Published:** 2025-12-08

**Authors:** E de Souza, D M D Mello, X Glaudas, E Hingst-Zaher, S M Almeida-Santos, C L Buck

**Affiliations:** Laboratório de Ecologia, Evolução e Conservação de Anfíbios e Répteis, Departamento de Ecologia, Universidade de São Paulo, Rua do Matão 321, Cidade Universitária, São Paulo, SP 05508-090, Brazil; Laboratório de Ecologia e Evolução, Instituto Butantan, Av. Vital Brasil 1500, Butantã, São Paulo, SP 05503-900, Brazil; Departamento de Fisiologia, Instituto de Biociências, Universidade de São Paulo, Rua do Matão 321 trav. 14, Butantã, São Paulo, SP 05508-090, Brazil; Laboratório de Ecologia, Evolução e Conservação de Anfíbios e Répteis, Departamento de Ecologia, Universidade de São Paulo, Rua do Matão 321, Cidade Universitária, São Paulo, SP 05508-090, Brazil; Museu Biológico, Instituto Butantan, Av. Vital Brasil 1500, Butantã, São Paulo, SP 05503-900, Brazil; Museu Biológico, Instituto Butantan, Av. Vital Brasil 1500, Butantã, São Paulo, SP 05503-900, Brazil; Laboratório de Ecologia e Evolução, Instituto Butantan, Av. Vital Brasil 1500, Butantã, São Paulo, SP 05503-900, Brazil; Department of Biological Sciences, Northern Arizona University, 617 S. Beaver St., Flagstaff, AZ 86011, USA

## Abstract

Understanding a species’ physiological state is important for advancing animal ecology and conservation. Endocrine responses to reproduction, stress, and nutritional status are commonly assessed through gonadal, adrenal, and thyroid hormones, respectively. Using nonconventional samples for endocrinological evaluation is an increasingly utilized method but remains uncommon for snakes. In this study, we assessed whether feces, urine, or shed skin from two Neotropical pit vipers (*Bothrops jararaca* and *B. jararacussu*) contain detectable testosterone (T), progesterone (P4), 17β-estradiol (E2), corticosterone (CORT), and triiodothyronine (T3) using enzyme immunoassay (EIA). We collected samples from 23 individuals, 10 *B. jararaca* and 13 *B. jararacussu*, and assessed detectability of hormones and/or immunoreactive hormone metabolites (IHM). We used tests of parallelism and accuracy to validate assays. Triiodothyronine was not detected in urine of either species; all other hormones were detected in all matrices. Testosterone and T3 showed good parallelism for all matrices tested. Parallelism tests for E2 (urine, both species), CORT (urine, *B. jararaca*, and shed skin, *B. jararacussu*), and P4 (urine, *B. jararaca*) showed marginally acceptable results. All accuracy validations were successful, except for T3 in shed skin extract (*B. jararacussu*) and P4 in urine extract (*B. jararaca*). This study demonstrates the applicability of nonconventional samples for hormone and IHM detection and quantification, offering valuable tools to monitor the endocrinological status of both free-ranging and confined snakes.

## Resumo (abstract in Portuguese)

Compreender o estado fisiológico dos animais é fundamental para o avanço da ecologia e conservação. As respostas endócrinas associadas a reprodução, estresse e status nutricional são geralmente avaliadas por meio de hormônios gonadais, adrenais e tireoidianos, respectivamente. O uso de amostras não convencionais para avaliação endó-crina é um método que tem sido cada vez mais utilizado, porém ainda pouco explorado em serpentes. Neste estudo, utilizamos ensaios imunoenzimáticos (EIA) para investigar se fezes, urina ou ecdise de duas espécies neotropicais de Viperidae (*Bothrops jararaca* e *B. jararacussu*) contêm níveis detectáveis de testosterona (T), progesterona (P4), 17β-estradiol (E2), corticosterona (CORT), e triiodotironina (T3). Coletamos amostras de 23 indivíduos, sendo 10 *B. jararaca* e 13 *B. jararacussu*, e analisamos a detectabilidade desses hormônios e/ou seus metabólitos hormonais imunorreativos (IHM). Utilizamos testes de paralelismo e acurácia para validar os ensaios. A triiodotironina não foi detectada na urina de ambas as espécies; o restante dos hormônios testados foram detectados em todas as matrizes. Testosterona e T3 apresentaram resultados satisfatórios de paralelismo para todas as matrizes testadas. Os testes de paralelismo para E2 (urina, ambas as espécies), CORT (urina, *B. jararaca*, e ecdise, *B. jararacussu*), and P4 (urina, *B. jararaca*), apresentaram resultados marginais aceitáveis. Testes de acurácia obtiveram resultados positivos, com exceção de T3 na ecdise de *B. jararacussu* e P4 na urina de *B. jararaca*. De forma geral, nossos resultados demonstram a aplicabilidade de amostras não convencionais para a detecção e quantificação de hormônios e IHM, validando ferramentas úteis para o monitoramento endocrinológico de serpentes de vida livre ou sob cuidados humanos.

## Introduction

The endocrine system of vertebrates is highly conserved (Campbell et al. 2004) and has evolved to modulate a myriad of physiological processes, such as reproduction, growth, and development (Dubois et al. 2002; Dufty et al. 2002; Gahete et al. 2009; Zwahlen et al. 2024). Across taxa, gonadal steroid levels often correlate with reproductive development and behavior (e.g., Buck and Barnes 2003; Remage-Healey and Bass 2006; Latham and Wade 2010), while increased glucocorticoid secretion typically reflects an adaptive response to stress (e.g., Denver 2009; Jessop et al. 2016; Herr et al. 2017; Polich 2018; Lind et al. 2018). Variation in circulating thyroid hormone concentration is associated with metabolism (Mullur et al. 2014), nutritional state, activity (e.g., Hulbert 2000; Joly et al. 2015; Wilstermann et al. 2015), and thermal acclimation (Little 2021). Therefore, knowing patterns of hormone secretion can provide key information into the life history, behavior, and physiological status of animals.

Traditionally, hormone quantification of both wild and captive animals has relied on blood samples (e.g., Wingfield and Farner 1976). However, sampling blood from free-living animals can be challenging for researchers and stressful to the animals, potentially altering physiological parameters (Sheriff et al. 2011; Lakušić et al. 2020). In addition, collecting blood for many species is not always possible due to conservation or ethical concerns (Kersey and Dehnhard 2014) or insufficient blood volume due to small body size (e.g., Dillon et al. 2023). Moreover, remote fieldwork often limits access to proper blood storage and transportation (Schilling et al. 2022). These challenges have driven the use of nonconventional sample types for extraction and quantification of hormones.

The collection of nonconventional sample types can offer the advantage of being minimally invasive, reducing or eliminating the need for capture, handling, or euthanizing individuals (Goymann 2005). Consequently, these samples are less susceptible to immediate stress-induced fluctuations in hormone concentrations (Buchanan and Goldsmith 2004; Sheriff et al. 2011). However, methods for measuring hormones extracted from nonconventional samples also have limitations. For example, hormones in fecal or urine samples are metabolized before excretion, and interspecies differences in metabolism and excretion must be considered (Palme et al. 2005). These factors, along with other matrix-specific characteristics, require that methods of extraction and quantification for each hormone or immunoreactive hormone metabolites (IHM) be validated for each new species, substrate, and target analyte (e.g., Goymann 2005; Sheriff et al. 2011; Dillon et al. 2023).

Despite challenges, the use of nonconventional sample types has become increasingly common in studies of wildlife conservation and physiological ecology (Goymann 2005). A wide range of matrices have proven to contain detectable hormones or IHMs in biologically meaningful concentrations, including feathers (e.g., Jenni-Eiermann et al. 2015; Branco et al. 2023), feces (Pirotta et al. 2023), urine (Scheun et al. 2018), baleen (Hunt et al. 2017; Lowe et al. 2022; Ajó et al. 2024), and shed skin of reptiles (Berkvens et al. 2013; Zena et al. 2022), among others. These studies describe or improve upon methods to extract and quantify hormones in nonconventional samples (e.g., Hunt et al. 2017), and assessed endocrinological patterns related to exposure to stressors (e.g., Ajó et al. 2024), reproduction (e.g., Lowe et al. 2022) or metabolism (e.g., Branco et al. 2023), highlighting the potential use of nonconventional sample matrices to identify physiological status in vertebrates.

Compared to the extensive research on mammals and birds, studies of reptile endocrinology using nonconventional samples remain scarce (but see Berkvens et al. 2013). Most existing knowledge comes from blood hormone measurements in a few temperate reptile species (Taylor and DeNardo 2011; Carbajal et al. 2024), with snakes being particularly underrepresented. However, applying the use of nontraditional sample matrices for extraction and quantification of hormone content from feces or shed skin of snakes may be especially valuable for studying small or endangered species (e.g., *Bothrops insularis*, Kasperoviczus et al. 2023), where blood sampling is often not feasible. Therefore, developing and validating these methods could improve our knowledge of snake physiology and, ultimately, inform decisions related to conservation actions, both *in situ* and *ex situ* (e.g., Kersey and Dehnhard 2014; Kasperoviczus et al. 2023).

We focused on two sympatric and relatively common pit vipers from southeastern Brazil, *Bothrops jararaca* and *B. jararacussu* (Martins et al. 2002; Nogueira et al. 2019). Reproduction in both species is markedly seasonal (Almeida-Santos and Salomão 2002; Silva et al. 2020). Mating coincides with early female vitellogenesis in the austral autumn–winter. Consequently, females store sperm in their reproductive tract until ovulation in spring, and parturition occurs during summer–autumn. In males, spermatogenesis occurs during spring and summer, before the mating season. Although the reproductive strategies of these species are known, the upstream endocrine modulation of the observed patterns of physiology and behavior remains unknown.

In this study, we validated enzyme immunoassays (EIAs) for detectability, parallelism, and accuracy for (i) reproductive steroid hormones (T, P4, and E2); (ii) the adrenal glucocorticoid (CORT); and (iii) the most active form of thyroid hormone (T3), using nonconventional sample types (shed skin, feces, and urine) from free-ranging and captive individuals of *B. jararaca* and *B. jararacussu*. Based on previous studies (e.g., Berkvens et al. 2013; Dillon et al. 2021; Zena et al. 2022), we expected both shed skin and feces to be suitable matrices for hormones and IHM extraction and detection. By developing and validating noninvasive methods for hormone monitoring in these species, we expect to provide tools to investigate the endocrine mechanisms underlying their seasonal reproductive patterns. Ultimately, this study could support future studies on the hormonal regulation of behavior, reproduction, and stress responses in wild snake populations.

## Methods

### Animals and sample collection

Animal collection, handling and all the procedures described below were performed under license from Instituto Chico Mendes de Conservação da Biodiversidade (SISBIO-ICMBio #81669-1) and the approval of the local animal ethics committee of the Instituto Butantan (CEUA #1912061221).

In 2022 and 2023, we collected samples from a total of 23 individuals (Table S1). Most (*n* = 16) were collected in the field at Fazenda do Etá (24°19′13″ S, 48°7′3″ W; municipality of Eldorado, São Paulo state, Brazil) for a radiotelemetry study and opportunistically sampled. The other six individuals were collected in the metropolitan region of the city of São Paulo, Brazil (see Table S1 for additional details), and kept in captivity for other studies. While in captivity, snakes had access to water *ad libitum* and were fed once per month with laboratory mice (10% of the snake body weight) and held indoors at 22–28ׄ°C, 55–70% humidity, and a 12:12 light/dark cycle.

All animals collected, both in the field and in captivity, were housed individually in 24 cm × 39 cm × 13 cm clear plastic boxes. For all individuals, we waited 24h for the snake to defecate or urinate to collect the sample. In reptiles, urine and feces are often excreted simultaneously but remain physically distinct. The white and solid appearance of the urate contrasts with the darker fecal matter, enabling researchers to separate the two sample types with minimal risk of cross-contamination (Kummrow et al. 2011). If a snake did not defecate or urinate within 24 h, we started the morphometric measurements. For all snakes, we measured snout–vent length (SVL; ±1 mm) and tail length (±1 mm) using a plastic ruler, and the total body mass using a spring scale (±0.3%, Balance Light Line, Pesola^®^, Switzerland). For individuals that did not spontaneously provide a sample within 24 h, we took advantage of the morphometric assessment to gently press the final third of the body of the snake to stimulate the release of urine or feces. The released sample was collected into a 50 mL polypropylene conical tube (Falcon tubes, K19-0050, Kasvi, Brazil) and kept frozen at –20°C until extraction. Urination was more frequent than defecation after stimulation.

Individuals were classified as mature or immature based on SVL (Sazima 1992; Silva et al. 2020), tail color patterns (Martins et al. 2002), and vent palpation of reproductive organs, a commonly used method to assess both vitellogenic follicles and embryos in females (e.g., Naulleau and Bonnet 1996; Stahl 2002; DeNardo 2004) and to stimulate the release of semen from males. We opportunistically collected skin samples from tagged animals tracked in the field.

For some animals (*n* = 4), we collected more than one sample because some snakes were being tracked or kept in captivity (see Table S1). In total, we collected nine fecal samples and five urine samples from *B. jararaca*, and four fecal samples, five shed skin samples, and five urine samples from *B. jararacussu*.

### Hormone extraction

We followed the extraction protocol of Graham et al. (1993, 2001) with minor modifications to the methanol concentration (90% instead of 80%) and volume of dilution (7 mL instead of 5 mL). All fecal, urine, and shed skin samples were individually homogenized. For fecal samples, we removed unwanted artifacts with a sieve, such as indigestible items (e.g., Racine et al. 2022). Resultant homogenized samples were freeze-dried (DIM Lyophiliser Enterprise series, v. 3.1) for 24 h and weighed. For each sample, 50 mg was mixed (BenchMixer™ V2, Benchmark Scientific Inc., Sayreville, NJ, USA) with 7 mL of 90% methanol and vortexed for 12 h (Benchmixer™ XL, Benchmark Scientific Inc., Sayreville, NJ, USA), and centrifuged for 20 min at 3000 *g* at room temperature (20 ± 2°C). The supernatant was pipetted into a glass tube and held in a laminar flow hood at room temperature until dry (approximately 24 h). Dried samples were reconstituted in 500 µL of EIA assay buffer (buffer X065, Arbor Assays, Ann Arbor, MI, USA) and vortexed for 2 h.

### Hormone assays

We performed colorimetric EIAs for T, P4, E2, CORT, and T3 using commercially available EIA kits from Arbor Assays (Ann Arbor, MI, USA, catalog numbers: K032-H, K025-H, K030-H, K014-H, and K056-H, respectively), which have been extensively tested to perform well using non-plasma samples from wildlife. A volume of 100 µL of each reconstituted sample from both species was combined to create five different full extract pools (“1:1”) representing all sample types and species assessed (i.e., *B. jararaca* feces = 900 µL, *B. jararaca* urine = 500 µL, *B. jararacussu* feces = 400 µL, *B. jararacussu* urine = 500 µL, and *B. jararacussu* shed skin = 500 µL). The 1:1 extract of each sample type and species was used for all assays and was serially diluted by progressive halving in EIA assay buffer, producing a total of eight dilutions per sample pool: 1:1, 1:2, 1:4, 1:8, 1:16, 1:32, 1:64, and 1:128. The dilutions were assayed in duplicate in all tests of detectability, and dilutions with detectable hormones were assayed in duplicate in all tests of parallelism and accuracy. All tests occurred within 3 months of extraction, and all dilutions were kept at –80°C during this period. We created additional dilutions (e.g., 1:256, 1:512, etc.) as deemed necessary by the detectability test. Assay protocols were followed as described by the manufacturer, with the exception of one minor modification: an additional low standard (25 pg/mL) was added to the P4 standard curve when assaying urine extracts.

All assays included duplicates of all nonspecific binding wells, blanks (zero dose), standards, and samples. Reported results reflect the average of the duplicates. Duplicates having a coefficient of variation >10% were reassayed. Intra-assay variation is available in Table S2.

#### Detectability

The detectability test demonstrates that the target hormone or IHM is present in the sample and detectable in the assay. We tested the detectability of the eight serial dilutions (1:1 to 1:128) for each combination of sample extracts and hormones or IHMs. The serial dilutions were assayed alongside known-dose hormone standards, comparing each sample type to the standard curve run in the same assay. For each combination of extract/hormone/species, we chose the dilution that fell within 20–50% binding to guide the parallelism tests performed subsequently.

#### Parallelism

The parallelism test demonstrates that serial dilutions of the sample result in a linear decrease in assay values that is parallel to the standard curve proportionally, suggesting that the sample behaves in a way that is immunologically similar to the standard and thus can be measured proportionally (Ezan et al. 2000). We conducted tests of parallelism for all combinations of sample extracts that had detectable hormone, using serial dilutions of our sample extracts, also assayed alongside known-dose hormone standards. Because T3 was not detectable in urine sample extracts of either species, we did not continue validating this combination of sample extract and hormone.

#### Accuracy

The accuracy assay evaluates potential matrix effects; that is, it determines whether components present in the sample extract interact with the assay system in a way that could lead to under- or overestimation of the actual hormone concentration (Ezan et al. 2000). The assay results are satisfactory when there is a linear relationship between concentrations of added and measured hormones (Ezan et al. 2000). For the accuracy tests, we used the most diluted concentration that had detectable hormone, that is, dilutions that fell between 60 and 80% of binding between antibody and labeled hormone. The selected dilution represents a region of the parallelism binding curve that has enough matrix to test its impact on the assay accuracy while there is not enough endogenous hormone to obscure the low concentration standards. For each accuracy test, we made a full standard curve, nonspecific binding wells, and a blank (zero dose) spiked in duplicate with an equal volume of appropriate dilution of sample extract, and the spiked standards were then assayed alongside a second standard curve that was spiked only with buffer (e.g., Hunt et al. 2017).

### Statistical analysis

Results of the tests of parallelism were graphed as a percentage of antibody bound *vs.* log of the relative dose. The resulting binding curves were assessed with an *F* test for difference in slope (indicated by *P*-value ≥ 0.05), with the linear portion of each sample extract binding curve compared to the standard curve that had been assayed on the same microplate (Grotjan et al. 1996; Ezan et al. 2000). Good assay performance is indicated by no significant differences in slope (*P* ≥ 0.05) between the linear portions of the binding curve of serially diluted samples and the standard curve. Results of the tests of accuracy were graphed as observed dose *vs.* expected standard dose and were assessed using linear regression. A slope close to 1.0 (acceptable range = 0.7–1.3) and a *R*^2^ close to 1.00 (acceptable *R*^2^ > 0.95) indicate that there is a linear relationship between concentrations of added and measured hormones, meaning that the assay can quantify hormones and distinguish high from low concentrations with acceptable mathematical accuracy (Grotjan et al. 1996; Ezan et al. 2000). We conducted the analysis and created graphics using the GraphPad Prism software (version 10.0.0 for Windows, GraphPad Software, Boston, Massachusetts, USA, www.graphpad.com).

## Results

### Detectability

Testosterone, E2, P4, and CORT were detectable in extracts of feces, urine, and shed skin from both *B. jararaca* and *B. jararacussu* (Table 1). Triiodothyronine was not detectable in urine extracts from either species. Thus, we did not proceed with T3 parallelism or accuracy validation for this sample matrix.

**Table 1:** Extracted sample dilutions providing 20–50% binding (ideal dilution) in assays of hormones from shed skin or IHM from urine and feces collected from *B. jararaca* and *B. jararacussu*

Species	Matrix	*T*	P4	E2	T3	CORT
*B. jararaca*	Feces	1:64	1:64	1:16	1:4	1:16
	Urine	1:4	1:4	1:2	ND	1:2
*B. jararacussu*	Feces	1:8	1:16	1:16	1:4	1:4
	Urine	1:8	1:4	1:2	ND	1:64
	Shed skin	1:4	1:16	1:2	1:2	1:2

*Note*: IHM of T3 was not detected (ND) in urine extracts from either species.

The dilution (extract: resuspension buffer) needed for hormone detection differed between species and among hormones and biological matrices. For example, for *B. jararacussu* shed skin, E2, T3, and CORT were detected at dilutions ranging from 1:1 to 1:8 for E2 and T3 and 1:1 to 1:16 for CORT. Similarly, *B. jararaca* urine required dilutions ranging from 1:1 to 1:8 for P4 and 1:1 to 1:16 for E2. In contrast, fecal samples detected IHM across a broader range of dilutions, from 1:1 to 1:128 for CORT, P4, and T3, and from 1:1 to 1:64 for E2 and T. In Table 2, we present the dilutions and respective percentage of binding within the linear portion of the standard curve (i.e., 20–50% of binding). These data indicate the ideal dilution for each hormone, biological matrix (extract), and species. We used the range of dilutions where hormones were detectable to complete the tests of parallelism and accuracy. In tests of parallelism and accuracy, we excluded dilutions where hormone/extract/species combinations were not detectable. The range of detectability for each combination of hormone/extract/species and the coefficient of intra-assay variation are available at Table S2.

**Table 2:** Results of *F*-tests for comparing slopes of the linear portions of the standard curve and the serial dilution of feces and urine extract for *B. jararaca* and feces, urine, and shed skin extract for *B. jararacussu* considering five enzyme immunoassays

Species	Matrix	T	P4	E2	CORT	T3
*B. jararaca*	Feces	*F*_1,8_ = 0.389 *P* = 0.55	*F*_1,6_ = 1.683 *P* = 0.24	*F*_1,6_ = 0.277 *P* = 0.62	*F*_1,8_ = 0.017 *P* = 0.90	*F*_1,4_ = 0.059 *P* = 0.82
	Urine	*F*_1,6_ = 1.226 *P* = 0.31	*F*_1,4_ = 1.703 *P* = 0.26	*F*_1,5_ = 5.380 *P* = 0.06	*F*_1,6_ = 4.254 *P* = 0.09	ND
*B. jararacussu*	Feces	*F*_1,8_ = 0.389 *P* = 0.55	*F*_1,6_ = 1.927 *P* = 0.21	*F*_1,4_ = 0.350 *P* = 0.59	*F*_1,8_ = 0.248 *P* = 0.63	*F*_1,6_ = 0.072 *P* = 0.80
	Urine	*F*_1,8_ = 1.199 *P* = 0.31	*F*_1,6_ = 5.741 *P* = 0.05	*F*_1,2_ = 20.870 *P* = 0.05	*F*_1,8_ = 0.560 *P* = 0.48	ND
	Shed skin	*F*_1,6_ = 0.520 *P* = 0.50	*F*_1,6_ = 0.009 *P* = 0.93	*F*_1,2_ = 5.817 *P* = 0.14	*F*_1,4_ = 6.419 *P* = 0.06	*F*_1,4_ = 0.175 *P* = 0.70

*Note*: Different degrees of freedom of each analysis are due to some dilutions having undetectable hormone content for some extracts (as explained in the “Detectability” section). Immunoreactive metabolites of T3 were not detected (ND) in urine extracts from either species.

### Parallelism

**Fig. 1:**
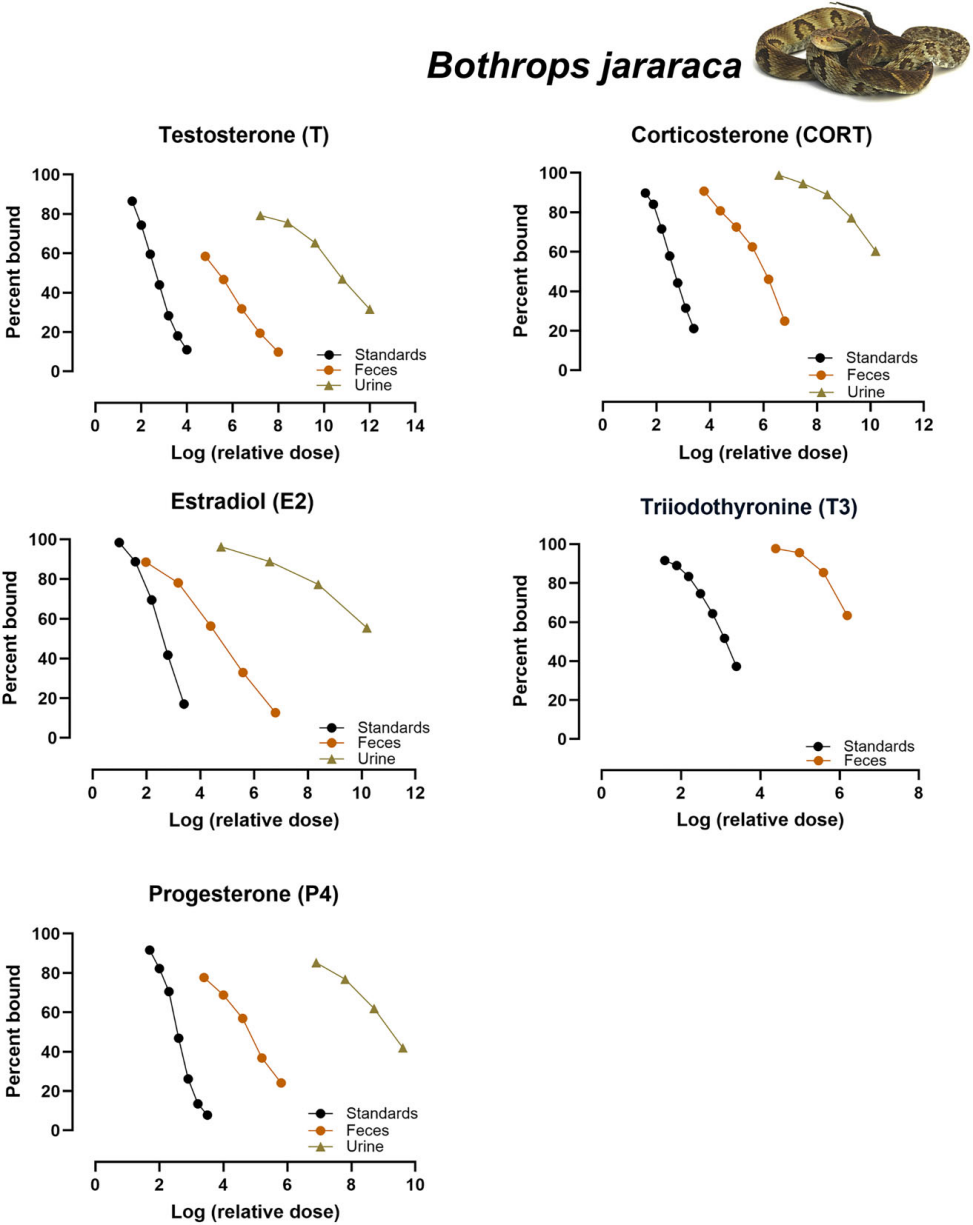
Results of parallelism tests for the five hormones (T, E2, P4, CORT, and T3) tested with serially diluted extracts of feces and urine from *Bothrops jararaca*. Only tests with *P*-values ≥ 0.05 are shown.

**Fig. 2:**
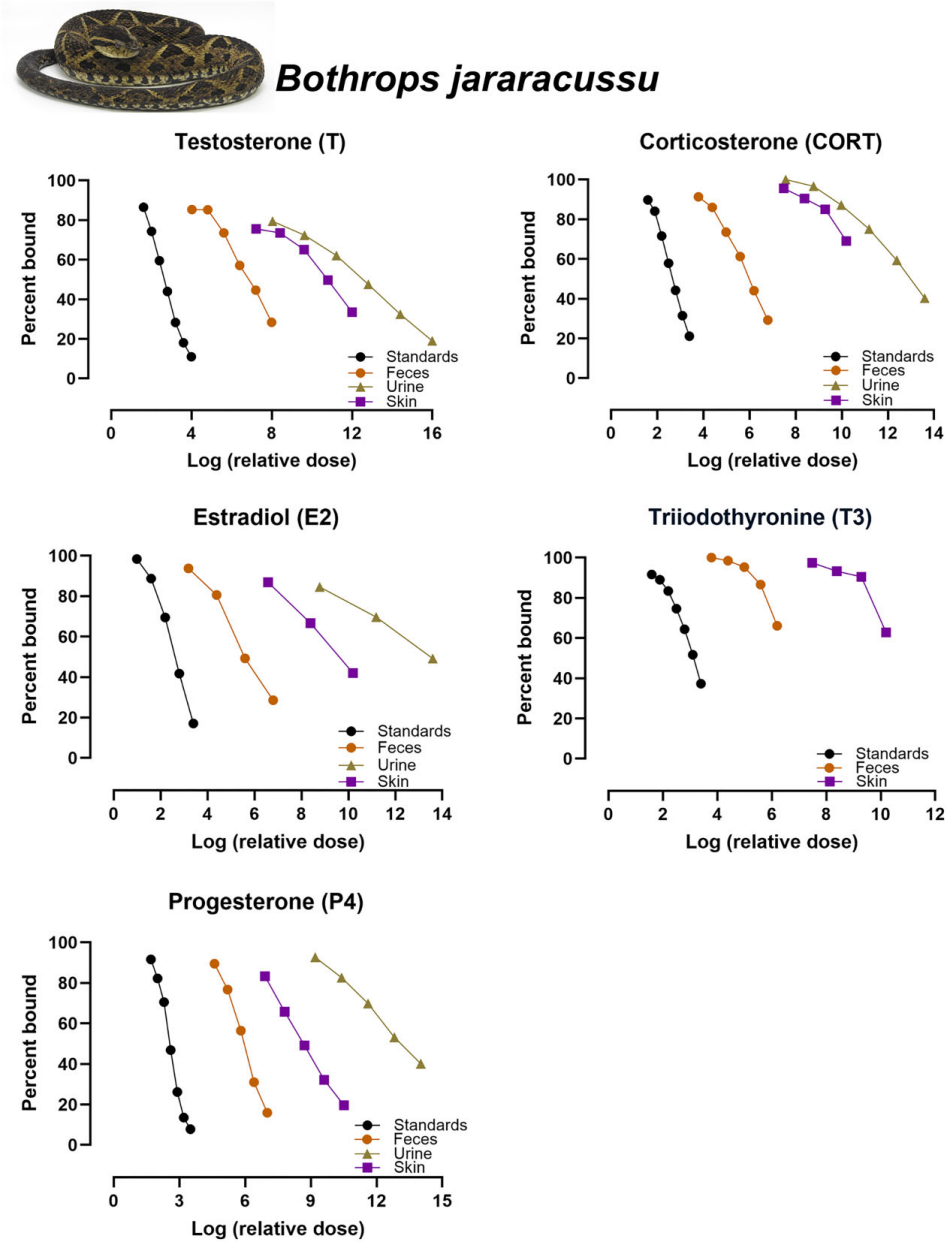
Results of parallelism tests for the five hormones (T, E2, P4, CORT, and T3) tested with serially diluted extracts of feces, urine, and shed skin from *Bothrops jararacussu*. Only tests with *P*-values ≥ 0.05 are shown.

Fecal extracts of both species showed acceptable parallelism for all IHM tested (Figs. 1 and 2; Table 2). Urine extracts from *B. jararaca* showed acceptable parallelism for T, P4, CORT (Fig. 1; Table 2), but for E2 it was at the threshold of acceptability, with *P* = 0.06. For *B. jararacussu*, urine extracts showed acceptable parallelism for T and CORT, and for P4 and E2 functioned at the threshold of acceptability, with *P* = 0.05 (Fig. 2; Table 2). Considering shed skin extracts, the CORT parallelism test also performed at the threshold of acceptability, with *P* = 0.06, and good for the other four hormones tested (Fig. 2; Table 2). The coefficient of intra-assay variation is available in Table S2.

### Accuracy

**Fig. 3:**
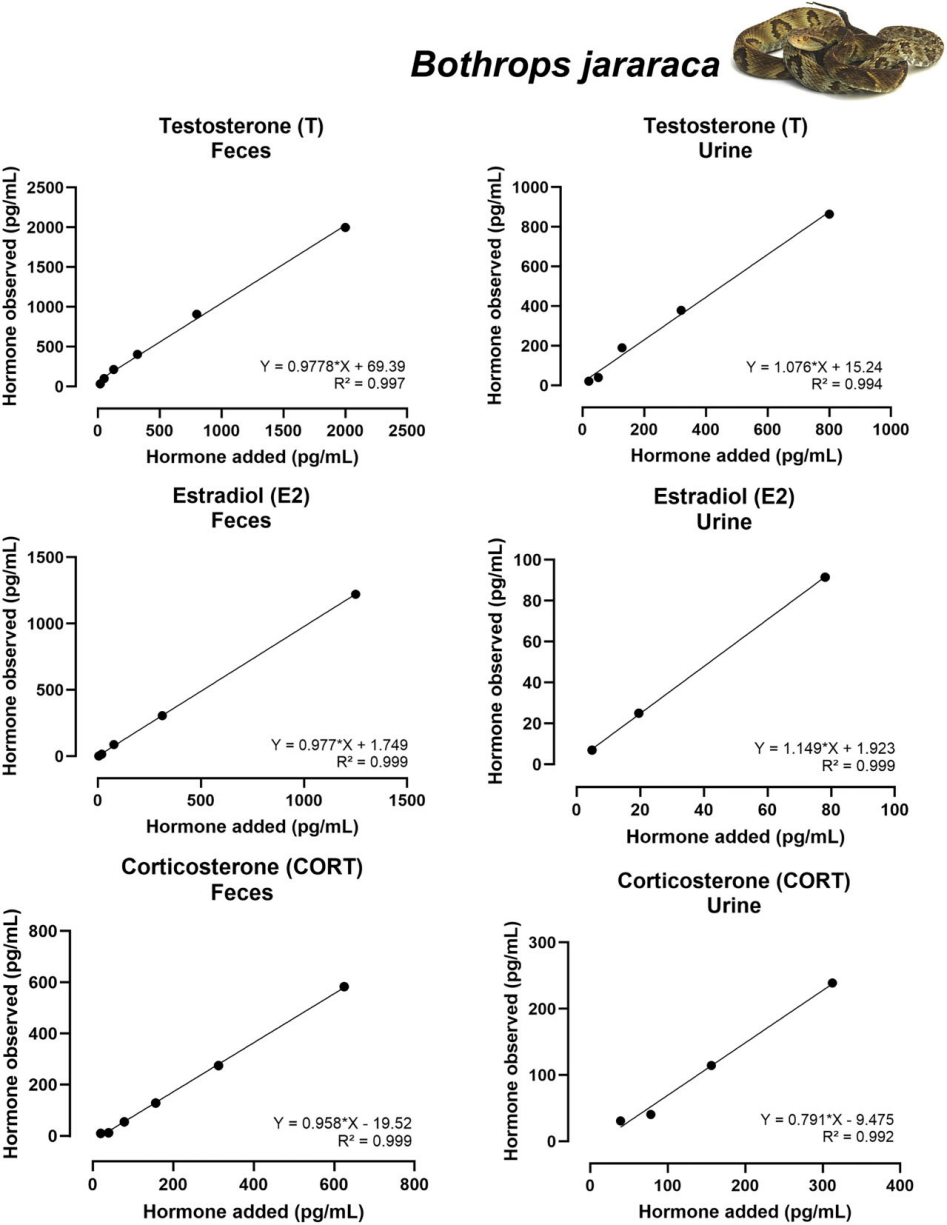
Results of accuracy tests for T, E2, and CORT tested with serially diluted extracts of feces (right) and urine (left) from *Bothrops jararaca*. Each panel shows the relationship between added and observed hormone concentrations (pg/mL). Equations of the linear regression and *R*² values are displayed in each panel. Only tests within the acceptable slope range of 0.7–1.3 are shown.

**Fig. 4:**
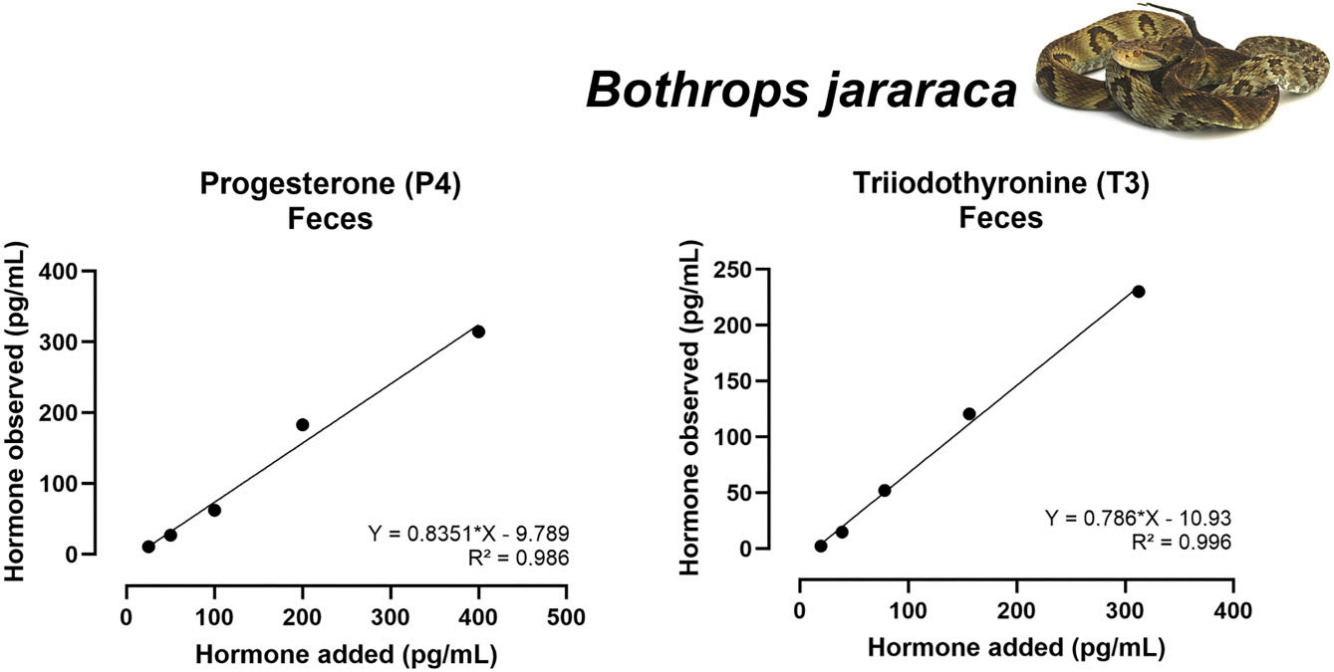
Results of accuracy tests for P4 (right) and T3 (left) tested with serially diluted extracts of feces from *Bothrops jararaca*. Each panel shows the relationship between added and observed hormone concentrations (pg/mL). Equations of the linear regression and *R*² values are displayed in each panel. Only tests within the acceptable slope range of 0.7–1.3 are shown.

**Fig. 5:**
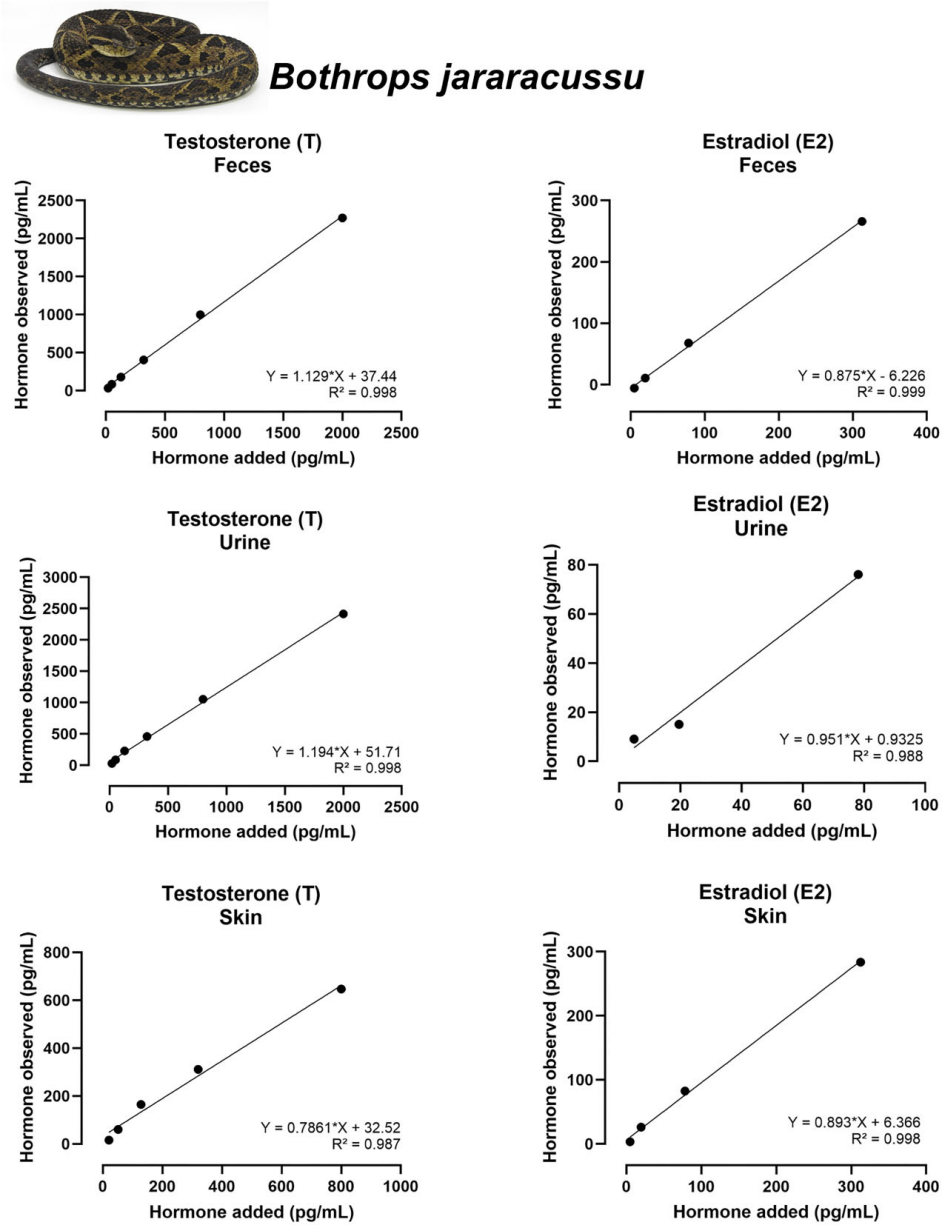
Results of accuracy tests for T (right) and E2 (left) tested with serially diluted extracts of feces, urine, and shed skin from *Bothrops jararacussu*. Each panel shows the relationship between added and observed hormone concentrations (pg/mL). Equations of the linear regression and *R*² values are displayed in each panel. Only tests within the acceptable slope range of 0.7–1.3 are shown.

**Fig. 6:**
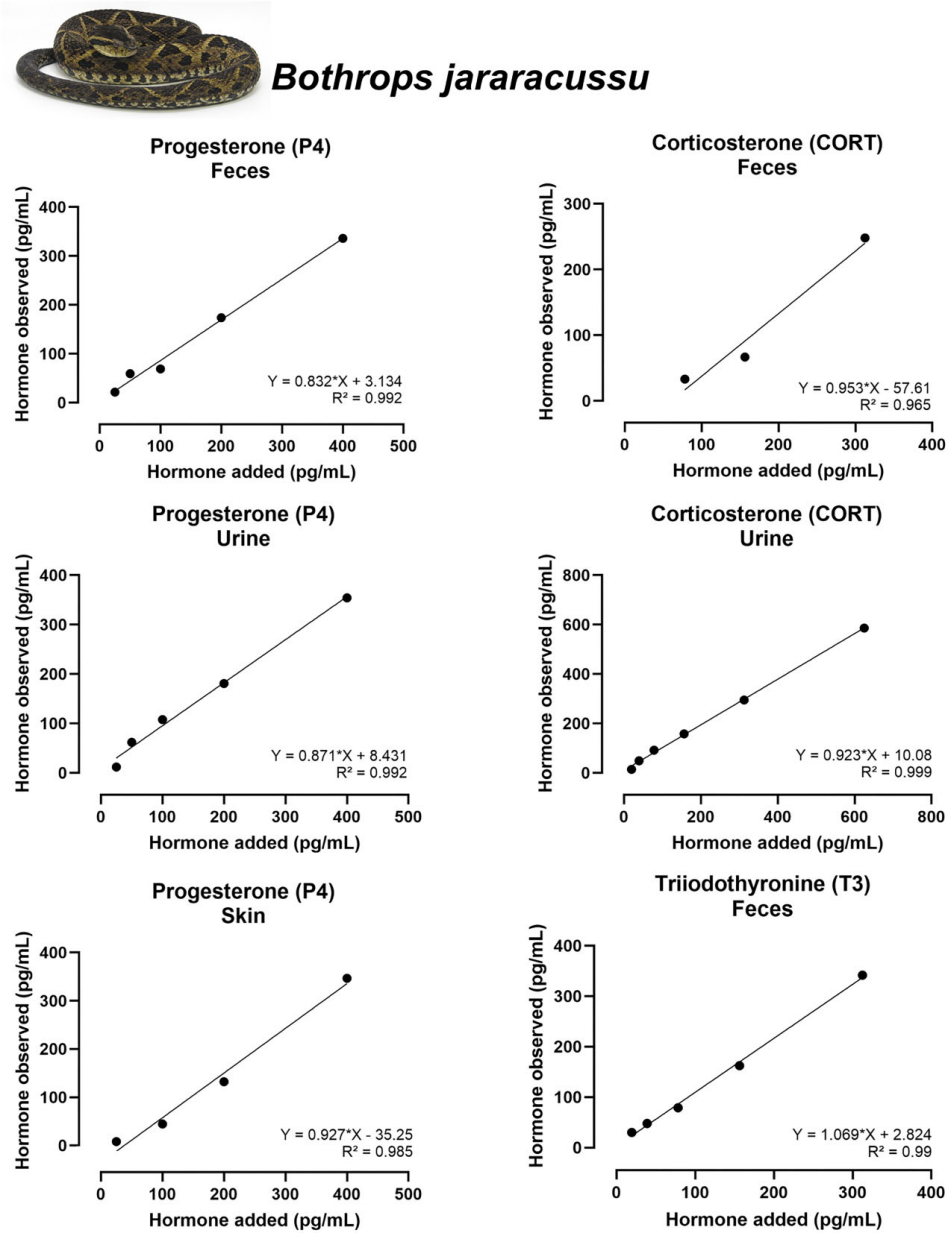
Results of accuracy tests for P4 (right), CORT (left), and T3 (left—bottom) tested with serially diluted extracts of feces, urine, and shed skin from *Bothrops jararacussu.* Each panel shows the relationship between added and observed hormone concentrations (pg/mL). Equations of the linear regression and *R*² values are displayed in each panel.

Most accuracy tests across combinations of the five hormones and three biological matrices yielded satisfactory linear relationships between observed and expected concentrations. Nine assays showed strong performance, with slopes between 0.9 and 1.1 (ideal = 1.0) and *R*^2^ ≥ 0.98: T (feces and urine of both species), E2 (feces and urine of both species and shed skin of *B. jararacussu*), CORT (feces of both speciesand urine of *B. jararacussu*), and T3 (feces of *B. jararacussu*) (Figs. 3–6; Table 3).

**Table 3:** Results of accuracy tests for all five EIAs performed with feces, urine, and shed skin extracts of *B. jararaca* and *B. jararacussu*

Species	Matrix	T	P4	E2	CORT	T3
*B. jararaca*	Feces	Slope = 0.978 *R*^2^ = 0.997	Slope = 0.835 *R*^2^ = 0.986	Slope = 0.977 *R*^2^ = 0.999	Slope = 0.958 *R*^2^ = 0.999	Slope = 0.786 *R*^2^ = 0.996
	Urine	Slope = 1.076 *R*^2^ = 0.994	Slope = 0.474* R^2^ = 0.986	Slope = 1.149 *R*^2^ = 0.999	Slope = 0.791 *R*^2^ = 0.992	ND
*B. jararacussu*	Feces	Slope = 1.129 *R*^2^ = 0.998	Slope = 0.832 *R*^2^ = 0.992	Slope = 0.875 *R*^2^ = 0.999	Slope = 0.953 *R*^2^ = 0.965	Slope = 1.069 *R*^2^ = 0.999
	Urine	Slope = 1.194 *R*^2^ = 0.998	Slope = 0.871 *R*^2^ = 0.992	Slope = 0.951 *R*^2^ = 0.988	Slope = 0.923 R^2^ = 0.999	ND
	Shed skin	Slope = 0.786 *R*^2^ = 0.987	Slope = 0.927 *R*^2^ = 0.985	Slope = 0.893 *R*^2^ = 0.998	ND	Slope = 1.531* R^2^ = 0.929

*Note*: The slopes of observed hormonal doses in standards spiked with all extracted matrices *vs*. unspiked standards are shown, along with the coefficient of determination (*R*^2^) of the linear regression line. * = Slopes > 1.3 and < 0.7 indicate that the hormone cannot be tested with mathematical accuracy in this assay due to matrix effects. IHM of T3 was not detected (ND) in urine extracts from either species; and CORT did not achieve acceptable accuracy (ND) in shed skin extracts from *B. jararacussu*.

Considering P4 assays, fecal extracts from both species exhibited acceptable accuracy (slopes = 0.835 and 0.832, respectively), as observed for urine and shed skin from *B. jararacussu* (slope = 0.871 and 0.927). Conversely, P4 accuracy for urine of *B. jararaca* was out of the acceptable range (slope = 0.474).

For T3, fecal extracts from both species showed acceptable accuracy (slopes = 0.786 for *B. jararaca* and 1.069 for *B. jararacussu*). The assay failed for shed skin of *B. jararacussu* (slope = 1.531, Table 3).

The CORT assays were satisfactorily accurate for fecal and urine extracts in both species (slopes between 0.791 and 0.958, and *R*² ≥ 0.965). However, for the shed skin assay, only two datapoints were available for regression testing, thus precluding meaningful statistical evaluation. Accuracy tests for T and E2 were acceptable for most assays, with slopes consistently within or close to the acceptable range and *R*² ≥ 0.99 (Table 3).

All accuracy tests that produced acceptable results are available in Table 3 and Figs. 3 and 4 for *B. jararaca*, and Figs. 5 and 6 for *B. jararacussu*. The coefficient of variation intra-assay is available at Table S2.

## Discussion

To assess the feasibility of using nonconventional matrices for endocrine monitoring in snakes, we assayed feces, urine, and shed skin samples from *B. jararaca* and *B. jararacussu*. We tested assay performance for hormones or IHM associated with the gonadal (T, P4, E2), adrenal (CORT), and thyroidal (T3) axes. The dilution ratios required for hormone detection and optimal binding varied among sample types. In snakes and other vertebrates, hormones, or IHM, accumulate in the gut (feces), ureters or bladders (urine), or epidermis (shed skin) during excreta formation or tissue growth. Thus, the hormone content of these sample types is thought to reflect the average circulating hormone levels over the period of formation (e.g., Sheriff et al. 2011; Cook 2012; Palme 2019), likely affecting the hormone concentrations in extracts (Sheriff et al. 2011). Also, factors like diet, hydration, or tissue growth might affect varied rates of analyte accumulation among different matrices (Palme et al. 2005; Scheun et al. 2018; Mayer et al. 2024). Therefore, when designing endocrine studies, one should take care in the selection of the most appropriate sample types to ensure that the hormone concentration reflects the timeframe of interest.

The extraction protocol can also be a source of variability in hormone and IHM detection and validation. Extraction efficiency may vary with sample size (e.g., Millspaugh and Washburn 2004) or sample material (e.g., Burgess et al. 2016). The final concentration of the hormone also reflects losses during the extraction process (Goymann 2005). Consequently, considerable effort has been devoted to developing techniques that maximize hormone recovery across species and sample types (e.g., Berkvens et al. 2013; Hunt et al. 2014). In this work, we applied a widely used protocol (Graham et al. 1993, 2001) to evaluate its efficiency in a broader context, considering multiple hormones, species, and matrices. Except for T3 in urine from both species, the extraction method yielded valid concentrations of the hormones and IHMs. However, the extraction process may have influenced CORT validation in shed skin samples of *B. jararacussu*. For example, Berkvens et al. (2013) tested a variety of extraction methods and successfully recovered CORT from shed skin of the African house snake (*Lamprophis fuliginosus*) and the Massasauga rattlesnake (*Sistrurus catenatus*), suggesting that controlling for extraction efficiency and performance should be tested in future studies.

In our tests of detectability and quantification of hormones and IHM’s, fecal samples performed better than urine and shed skin. Previous studies have shown that quantifying IHM content in fecal samples is an appropriate method for endocrine research in snakes. For example, fecal CORT concentrations positively correlated with blood plasma levels in the African house snake (Berkvens et al. 2013) and in the common gartersnake (*Thamnophis sirtalis*; Halliday et al. 2015), indicating that fecal samples can be used as an alternative for blood samples. Further, fecal IHM levels have been shown to behave as expected in other snake species: P4 levels fluctuate during the reproductive cycle of ball pythons (*Python regius*) and show a peak in metabolite concentration during pregnancy (Bertocchi et al. 2018); and an increase in fecal CORT metabolite occurs in response to stressors in Wagner’s viper (*Montivipera wagneri*) (Augustine et al. 2022). Our study builds on our current knowledge of fecal IHM use in reptiles by introducing methods to detect and quantify T and T3 in snake’s feces for the first time and by validating CORT, P4, and E2 for two new species, *B. jararaca* and *B. jararacussu*.

Fecal IHM content is likely influenced by factors such as feeding frequency and fecal retention time. In snakes, fecal retention time is species-specific and correlates with body mass, with larger snakes retaining feces longer (Lillywhite et al. 2002). Feeding frequency also varies within the group. *Bothrops* snakes, for example, typically rely on ambush hunting (Martins et al. 2002), which likely results in infrequent feeding, feces formation, and defecation. In contrast, captive snakes are fed regularly, which may allow for systematic collection and longitudinal analysis of IHM concentrations (e.g., Augustine et al. 2022). In our study, two individuals of *B. jararaca* kept in captivity defecated 19 and 13 days after feeding, respectively. However, it is unclear whether these defecations were directly related to the most recent feeding or an earlier period of digestion and feces formation. The African house snakes kept in captivity (Berkvens et al. 2013) defecated at intervals ranging from a few days to several weeks, reinforcing the variability in fecal formation timing among snakes. We recommend researchers validate time lags between hormone content and feces formation in their target species. Such validations may include using labeled hormones or nontoxic colored craft chips (e.g., Palme et al. 2005; Kummrow et al. 2011). After biological and methodological validation, fecal IHM content can be reliably interpreted.

The shed skin matrix also showed promising assay performance, with all hormones detected and consistently quantifiable across dilutions, as indicated by tests of parallelism. However, CORT and T3 assays of shed skin did not achieve acceptable accuracy, likely due to extraction protocol or interference from keratin or other compounds in the matrix. Dillon et al. (2021) explored using keratinase, an enzyme that breaks down keratin, to increase hormone extraction efficiency across species and sample types. Keratinase treatment was ineffective for shed skin of the Argentine black and white tegu (*Salvator merianae*), but it improved CORT yield in shed skin of the narrow-headed garter snake (*Thamnophis rufipunctatus*). Given the need to validate each hormone and species, we suggest further testing of keratinase for snake shed skin in other species, including *Bothrops*. Further, the timing of skin formation must be considered. Shedding frequency in snakes varies with factors such as body/environmental temperature, injury, feeding, and reproduction (Smith and Barker 1988; Gibson et al. 1989; Greene et al. 2002; Carlson et al. 2014; Carnes-Mason and Beaupre 2023). In a 25-year study with a natural population of western diamondback rattlesnake (*Crotalus atrox*), individuals shed once or twice per year (Carnes-Mason and Beaupre 2023). Similarly, we observed one to three sheds annually in free-ranging *B. jararacussu* (*pers. obs.*, EdS and XG). Thus, shed skin may not be appropriate for addressing short-term hormonal fluctuations, such as those tied to seasonal cycles, particularly in wild populations.

Research on hormone detection in shed skin of snakes and lizards is sparse and has yielded mixed results. Studies with lizards have found meaningful CORT variation across sex, age, and season (e.g., Carbajal et al. 2018; Zena et al. 2022), but snake studies have failed to detect CORT differences under experimental or natural conditions (Berkvens et al. 2013; Mayer et al. 2024). This discrepancy may reflect the extended period over which hormones accumulate in growing skin, potentially masking short-term fluctuations in hormone levels. Alternatively, the permeability of thin skin tissues could allow for post-growth hormone penetration, reflecting more recent endocrine status (Zena et al. 2022). Our study contributes to this growing field by detecting T, P4, E2, CORT, and T3 in the shed skin of *B. jararacussu*, and we suggest further investigation on the complexity of hormone accumulation in shed skin, and biological validation of the frequency of shed formation for assessing endocrine profiles in snake populations.

Urine presents a promising matrix for monitoring short-term hormone fluctuation in free-ranging or captive snakes. All IHMs tested, except for T3, were detectable in the urine of *B. jararaca* and *B. jararacussu* with optimal extract: resuspension concentrations ranging from 1:2 to 1:64. Although we are unaware of any published data on urination frequency in snakes, our observations indicate that *B. jararaca* urinates approximately once per week and with feces. In free-ranging individuals, we collected urine samples more frequently than fecal samples. This higher frequency of urination is important for longitudinal endocrine studies, as it allows for more consistent IHMs monitoring over time.

However, our results indicate that validation of IHMs in urine requires further refinement before broad implementation. The detected IHMs showed varied performance in validation tests of parallelism and accuracy. The parallelism test for E2 in *B. jararaca* and for E2 and P4 in *B. jararacussu* was at the threshold of acceptability and should be interpreted with caution. The accuracy test for P4 in *B. jararaca* showed a slope <0.7, likely due to matrix effects. In other taxa, urine dilution is often corrected using endogenous markers such as creatinine (e.g., Germano et al. 2012). Correction factors can improve assay performance by normalizing hormone metabolite levels relative to urine volume, ensuring that IHM concentrations reflect actual physiological states rather than fluctuating water content. However, in a study on the lizard *Smaug giganteus*, the authors did not apply any correction factor and observed biologically meaningful IHM fluctuations (Scheun et al. 2018). We recommend further studies on how IHM are incorporated during the urine formation in snakes to assess whether correction factors are necessary for accurate hormone metabolite analysis.

*Bothrops* species share conserved reproductive traits, including an autumn mating season, obligatory female sperm storage, seasonal parturition, and female-biased sexual size dimorphism (Almeida-Santos and Salomão 2002). *Bothrops jararaca*, the closest continental relative to several island-endemic and threatened species (Alencar et al. 2016), can serve as a model for understanding reproductive cycles and endocrine regulation, helping to identify conserved patterns within the genus and guiding research on other congeners. *Bothrops jararacussu*, one of the largest lancehead species and with the most accentuated female-biased sexual size dimorphisms in pit vipers (Silva et al. 2020), provides a unique opportunity to explore hormonal underpinnings of the evolution of dimorphism in snakes (Wilwert and Stuginski 2025). Our study highlights methodological recommendations, refinements, and best practices related to each specific characteristic of the tested matrices, such as formation period and frequency of production. Our validated approach supports broader applications in endocrine studies, offering new tools for investigating hormone dynamics in *Bothrops* species under natural conditions or in captivity.

## Supplementary Material

obaf048_Supplemental_Files

## Data Availability

The data supporting this work is available throughout the text and in Supplementary material.
